# Introducing a Thermo-Alkali-Stable, Metallic Ion-Tolerant Laccase Purified From White Rot Fungus *Trametes hirsuta*

**DOI:** 10.3389/fmicb.2021.670163

**Published:** 2021-05-21

**Authors:** Jing Si, Hongfei Ma, Yongjia Cao, Baokai Cui, Yucheng Dai

**Affiliations:** Institute of Microbiology, School of Ecology and Nature Conservation, Beijing Forestry University, Beijing, China

**Keywords:** laccase (Lac), white rot fungi (WRF), enzymatic performance, endocrine disrupting chemicals (EDC), bioremediation

## Abstract

This study introduces a valuable laccase, designated ThLacc-S, purified from white rot fungus *Trametes hirsuta*. ThLacc-S is a monomeric protein in nature with a molecular weight of 57.0 kDa and can efficiently metabolize endocrine disrupting chemicals. The enzyme was successfully purified to homogeneity via three consecutive steps consisting of salt precipitation and column chromatography, resulting in a 20.76-fold increase in purity and 46.79% yield, with specific activity of 22.111 U/mg protein. ThLacc-S was deciphered as a novel member of the laccase family and is a rare metalloenzyme that contains cysteine, serine, histidine, and tyrosine residues in its catalytic site, and follows Michaelis-Menten kinetic behavior with a *K*_*m*_ and a *k*_*cat*_/*K*_*m*_ of 87.466 μM and 1.479 s^–1^μM^–1^, respectively. ThLacc-S exerted excellent thermo-alkali stability, since it was markedly active after a 2-h incubation at temperatures ranging from 20 to 70°C and retained more than 50% of its activity after incubation for 72 h in a broad pH range of 5.0–10.0. Enzymatic activities of ThLacc-S were enhanced and preserved when exposed to metallic ions, surfactants, and organic solvents, rendering this novel enzyme of interest as a green catalyst for versatile biotechnological and industrial applications that require these singularities of laccases, particularly biodegradation and bioremediation of environmental pollutants.

## Introduction

Enzymes are currently receiving considerable interest from the public due to their impressive catalytic functionality, relatively broad abundance, and environmentally friendly nature (Buchholz and Bornscheuer, 2017; Wiltschi et al., 2020). Laccase (*p*-diphenol: dioxygen oxidoreductase, EC1.10.3.2) is a well-studied enzyme belonging to a family of polyphenol oxidases that have a copper-containing catalytic core, are widely distributed in fungi, bacteria, insects, and plants, and are classified based on molecular complexity and biological distribution (Thurston, 1994; Claus, 2004; Baldrian, 2006; Mate and Alcalde, 2015). Laccases of white rot fungi origin are ubiquitously superior to other enzymes in terms of lignin degradation. Thus, a series of laccases obtained from white rot fungi have attracted significant interest (Baldrian, 2006; Rivera-Hoyos et al., 2013; Chen et al., 2019). Laccases are involved in the metabolism of a multifarious range of aromatic compounds, organic pollutants, and inorganic substrates, with a concomitant four-electron reduction process that only generates water as a by-product. Consequently, these enzymes have huge potential as green catalysts in the fields of pulping and papermaking, biosynthesis, food processing, biosensor manufacturing, and bioconversion and refinement of agricultural and forestry wastes, in particular biodegradation and bioremediation of environmental contaminants (Giardina et al., 2010; Pezzella et al., 2015; Senthivelan et al., 2016; Bilal et al., 2019). To keep pace with rapidly increasing industrial demands, it is imperative to capture laccases capable of tolerating high temperature, alkali pH, the existence of metallic ions and chemical reagents, or other harsh environments from high-producing and easily available white rot fungal repositories (Si et al., 2013; Zheng et al., 2017; Liu et al., 2020).

Endocrine disrupting chemicals (EDCs), which are widespread in eco-environments and used in large amounts globally, are a category of emerging, highly toxic pollutants that include naturally produced compounds such as estrogens, androgens, and phytoestrogens, as well as various industrial chemicals and household products such as synthetic hormones, polycyclic aromatic hydrocarbons (PAHs), and pharmaceuticals (Darbre, 2019; Kasonga et al., 2021). These compounds can interfere with hormonal systems and adversely impact sexual development, reproduction, the nervous system, and immunity of both wildlife and humans, even at concentrations as low as parts per million (ppm) and parts per billion (ppb) (Su et al., 2020; Viguié et al., 2020). For example, 17β-estradiol (E2) is a representative estrogen that causes serious environmental and global issues owing to its induction of reproductive disorders, immune deficiencies, and even carcinogenic risks (Ye et al., 2017; Du et al., 2020).

This study aimed to purify an extracellular laccase ThLacc-S from white rot fungus *Trametes hirsuta* and obtain data on its enzymatic performance to scrutinize its functional involvement in bioremediation of EDCs and potential use as a green catalyst in industrial and biotechnological applications.

## Materials and Methods

### Chemicals

All chemicals used in this study were of analytical reagent grade. E2 was prepared by filtration through a 0.22-μm membrane to remove bacteria. Cetyl trimethyl ammonium bromide (CTAB) rapid plant genome extraction kits-DN14 were purchased from Aidlab Biotechnologies Co., Ltd, Beijing, China. Agar, 2,2’-azino-bis(3-ethylbenzothiazoline-6-sulfonic acid) (ABTS), bovine serum albumin (BSA), diethylaminoethyl (DEAE)-Cellulose ionic exchange column packing, Sephadex G-100 column packing, β-mercaptoethanol, guaiacol, trypsin, iodoacetic acid (IAA), tosyl-L-lysine chloroethyl ketone (TLCK), phenylmethylsulfonyl fluoride (PMSF), diethylpyrocarbonate (DEP), *N*-acetylimidazole (NAI), and ethylenediaminetetraacetic acid (EDTA) were all Sigma-Aldrich (St. Louis, MO, United States) reagents.

### Fungal Strain and Culture Conditions

A strain of *T. hirsuta* was isolated from a harvested fruiting body and cultivated on malt yeast agar (MYA) slants (g/L ultrapure water: malt extract 5, glucose 20, agar 20, KH_2_PO_4_ 1, MgSO_4_⋅7H_2_O 0.5, ZnSO_4_⋅7H_2_O 0.1, CuSO_4_⋅5H_2_O 0.1, and vitamin B1 0.01) for 7 days at 28°C. When mycelia covered the MYA slants, cultures were stored at 4°C and were sub-cultured once every 2 months.

### Ribosomal DNA (rDNA) Sequencing Analysis

Identification of the *T. hirsuta* isolate was carried out by sequencing analysis of the internal transcribed spacer (ITS) rDNA. Mycelia from overgrown MYA plates covered with cellophane membrane were harvested for total genomic DNA extraction using the improved CTAB protocol (Cui et al., 2019) with some modifications. Regions of ITS-rDNA (ITS-1, 5.8S, and ITS-2) were amplified by PCR using the universal primers ITS5 (5′-GGAAGTAAAAGTCGTAACAAGG-3′) and ITS4 (5′-TCCTCCGCTTATTGATATGC-3′). The PCR program comprised 95°C for 3 min, followed by 34 cycles of denaturation (94°C, 40 s), primer annealing (54°C, 45 s), and extension (72°C, 1 min), then a final 10-min extension at 72°C followed by cooling to 4°C. Amplification products were sequenced and compared with those in the GenBank database using the National Center for Biotechnology Information (NCBI)-BLAST tool.

### Inoculum Preparation and Flask Fermentation Cultivation

To prepare seed inoculum, the maintained strain was initially activated on MYA in a Petri dish for 6 days. Five 1-cm^2^ areas of the agar culture were chipped off with a sterilized perforator and transferred into a 250-mL Erlenmeyer flask containing 100 mL liquid malt yeast medium (MY, identical to MYA without agar). After cultivation for 6 days at 28°C on a rotary shaker at 150 rpm, the culture broth was mildly homogenized with a blender at 5,000 rpm for 1 min then used as seed inoculum.

Flask fermentation experiments were performed in 250-mL Erlenmeyer flasks containing 100 mL MY medium inoculated with 10 mL seed inoculum and cultivated at 28°C on a rotary shaker at 150 rpm for 6 days. Subsequently, mycelia and cell debris in the fermentation broth were removed by centrifugation (12,000 rpm, 20 min) and the resulting cell-free supernatant was deemed an extracellular enzyme source for further purification and was designated ThLacc.

### ThLacc Activity Assay and Protein Quantification

A ThLacc activity assay was implemented quantitatively as described by Si et al. (2013), based on colorimetric measurement at 420 nm by monitoring the change in absorbance due to oxidation of the substrate ABTS. One unit of laccase activity is the amount of enzyme required to cause one absorption increase per minute per milliliter of reaction mixture under assay conditions. A Bradford assay was used to evaluate protein concentration at 280 nm using BSA as a standard (Bradford, 1976).

### ThLacc Purification Procedure

Initially, the cell-free supernatant containing laccase ThLacc, seized through flask fermentation cultivation, was saturated up to 75% with solid (NH_4_)_2_SO_4_ under constant stirring at 4 °C then under stationary conditions overnight. The precipitate recovered after centrifugation at 12,000 rpm for 20 min at 4°C was suspended in 0.1 M citrate-phosphate buffer (pH 5.0) and dialyzed overnight against repeated changes of ultrapure water.

The isolated retentate was chromatographed onto a DEAE-Cellulose ionic exchange column (27 × 350 mm) pre-equilibrated with 0.1 M citrate-phosphate buffer (pH 5.0). The column was extensively washed-out with the citrate-phosphate buffer at a flow rate of 3.0 mL/min to remove unbound proteins until the absorbance at 280 nm was < 0.05. A linear gradient concentration of NaCl from 0.0 to 1.0 M at a flow rate of 1.0 mL/min was used for stepwise elution of the bound fractions, collecting 5.0 mL eluate/tube. Fractions were then examined for enzymatic activity and protein content. All fractions exhibiting laccase activity were pooled in their respective peak, dialyzed, and concentrated by ultrafiltration with a 50-kDa cut-off.

The fraction with highest laccase activity was subsequently gel-filtered on a Sephadex G-100 column (27 × 600 mm) pre-balanced with the citrate-phosphate buffer. Elution from the column was achieved with the same buffer at a flow rate of 1.0 mL/min. Fractions were collected (5.0 mL/tube) and the absorbance was detected at wavelengths of 420 and 280 nm; fractions with laccase activity were pooled, dialyzed, and concentrated for further study.

### Electrophoresis, Mass Spectrometry, and Amino Acid Sequencing

Uniformity and subunit molecular weight (Mw) of ThLacc-S was authenticated using denaturing sodium dodecyl sulfate-polyacrylamide gel electrophoresis (SDS-PAGE), composed of a 12% (w/V) separating gel (pH 8.8) and a 5% (w/V) stacking gel (pH 6.8), as portrayed by Laemmli (1970). A pre-stained protein marker mixture with apparent Mws from 10 to 100 kDa was applied for calibration, and protein bands formed in gels were visualized by staining with Coomassie Brilliant Blue R250.

To reflect the presence of laccase, native PAGE was deployed in a similar manner as SDS-PAGE without the addition of SDS and reducing reagent (β-mercaptoethanol) and without boiling the protein sample. After electrophoresis, the gel was washed with ultrapure water and subjected to a staining solution of 1.0 mM ABTS or guaiacol until colorized bands appeared.

Native Mw was unearthed by comparing the elution volume of ThLacc-S with reference proteins passed to a Sephadex G-100 gel filtration column and eluted with 0.1 M citrate-phosphate buffer (pH 5.0) at a flow rate of 1.0 mL/min. Reference protein markers used for calibration were aldolase (158.0 kDa), conalbumin (75.0 kDa), BSA (67.0 kDa), ovalbumin (43.0 kDa), carbonic anhydrase (29.0 kDa), ribonuclease A (13.7 kDa), and aprotinin (6.5 kDa).

For *N*-terminal amino acid sequencing, purified ThLacc-S was run on native PAGE then transferred to trypsin digestion. After staining with ABTS, the blotted protein band was excised, destained, and sent for sequencing to identify the protein of interest. The Mw of ThLacc-S was also determined by matrix-assisted laser desorption/ionization time-of-flight mass spectrometry (MALDI-TOF MS; AB SCIEX, United States) with a MALDI matrix composed of α-cyano-4-hydroxycinnamic acid. Mass spectra were processed through the MASCOT search engine (Matrix Science, United Kingdom) and laccase sequences were retrieved using the NCBI-BLAST database to search for fungal laccases with a high level of sequence similarity. Homologous sequences were aligned by using the ClustalX1.83 algorithm and DNAMAN6.0 software.

### Determination of Enzymatic Performance

The optimum pH where ThLacc-S exerted greatest activity was explored via an enzymatic assay at 25°C using ABTS as the substrate and adjusting the pH from 1.0 to 13.0 in increments of 1.0 pH unit with various buffers. These buffer systems included glycine-HCl buffer (pH 1.0–3.0), citrate-phosphate buffer (pH 3.0–5.0), 2-(*N*-morpholino)ethanesulfonic acid buffer (pH 5.0–6.0), phosphate buffer (pH 6.0–8.0), Tris-HCl buffer (pH 8.0–9.0), glycine-NaOH buffer (pH 9.0–11.0), Na_2_HPO_4_-NaOH buffer (pH 11.0–12.0), and KCl-NaOH buffer (pH 12.0–13.0). The pH stability of ThLacc-S was estimated from pH 1.0 to 13.0 by pre-incubating the purified enzyme at 25°C for 72 h. ThLacc-S activity in response to temperature was assessed between 10 and 90°C, in increments of 5°C, by incubating the enzyme at optimum pH with ABTS as the substrate. Thermostability of ThLacc-S was determined by pre-incubating the enzyme for 2 h at the aforenoted temperature ranges and conditions. Aliquots of samples were taken for measurement of remaining laccase activity toward ABTS and were expressed as percentage to highest activity.

ThLacc-S activity in the presence of various metallic ions (Li^+^, Na^+^, Mg^2+^, Al^3+^, K^+^, Ca^2+^, Mn^2+^, Fe^2+^, Fe^3+^, Ni^2+^, Cu^2+^, Zn^2+^, Pb^2+^, Ag^+^, Cd^2+^, Ba^2+^, or Hg^2+^) at final concentrations of 25.0 mM was investigated. Specific inhibitors of enzymatic activity such as IAA (cysteine protease inhibitor), TLCK (lysine protease inhibitor), PMSF (serine protease inhibitor), DEP (histidine protease inhibitor), NAI (tyrosine protease inhibitor), pepstatin A (aspartate protease inhibitor), and EDTA (chelator), surfactants such as SDS (ionic surfactant) and Triton X-100 (non-ionic surfactant), and organic solvents such as methanol, ethanol, propanol, hexane, acetone, toluene, and chloroform, were assayed at different concentrations for their influences on ThLacc-S activity. Assays were conducted by pre-incubating ThLacc-S with the respective additive at pre-selected concentrations at 50°C for 20 min, followed by evaluation of residual activity using the standard method with ABTS as the substrate. A control using ultrapure water instead of additives was accepted as 100% relative activity.

The Michaelis-Menten constant (*K*_*m*_) and catalytic constant (*k*_*cat*_) of ThLacc-S were determined, by using ABTS as a substrate at various concentrations ranging from 0.1 to 1.0 mM in 0.1 M citrate-phosphate buffer (pH 5.0) at optimum pH and temperature conditions. A Lineweaver-Burk plot was applied for calculation of kinetic parameters by linear regression.

### Detection of EDC-Bioremediating Capacity

Bioremediation of EDCs by ThLacc-S was achieved in a 10.0-mL mixture of 1.0 M citrate-phosphate buffer (pH 5.0) containing 5.0 U/mL purified enzyme solution and 0.01 mM E2. The mixture was treated dynamically at 55°C for 72 h in darkness under a shaking speed of 150 rpm. Control groups with inactivated ThLacc-S or EDC-free reactions were set at the same conditions. At desired time intervals, reactions were stopped by adding 10.0 mL methanol and centrifuging at 12,000 rpm for 20 min. The resulting supernatants were concentrated by rotary vacuum evaporator and used for gas chromatography-mass spectrometry (GC-MS) analysis to identify partial by-products of E2 metabolized by ThLacc-S. Aliquots (0.1 mL) of supernatant were injected into a Shimadzu QP2010-SE GC-MS Spectrometer (Shimadzu, Japan) equipped with an ionization detector using a Resteck column (0.25 × 30 nm, XTI-5). The mobile gas was ultrapure helium gas at a flow rate of 0.7 mL/min in a linear 30-min run time. The initial column temperature was held at 70°C for 2 min, then subjected to an increase of 10°C/min up to 280°C, and finally held at 280°C for 9 min. Chemical formulas of the possible metabolic by-products were clarified based on mass spectra and retention times on their gas chromatographs.

### Statistical Analysis

This study was conducted with completely randomized experimental designs. All determinations were performed in triplicate. Data comparison was statistically computed by analysis of variance (ANOVA) followed by Waller-Duncan test using SPSS 20.0 software.

## Results and Discussion

### Purification of ThLacc

Sequencing analysis was used for identification of the fungal isolate based on amplification of the ITS5 and ITS4 regions. A BLAST search of the ITS-rDNA sequence of the isolate compared with those in the GenBank database suggested 99% similarity with *T. hirsuta*, thereby verifying that the isolate was white rot fungus *T. hirsuta* (GenBank accession number: MW881532).

Extracellular laccase ThLacc from *T. hirsuta* was purified to homogeneity via three consecutive procedures consisting of (NH_4_)_2_SO_4_ saturation (5–100%) followed by ionic exchange and gel filtration chromatography. A summary of all purification data is tabulated in Table 1. Culture supernatant containing laccase (1.065 U/mg protein) was firstly precipitated with 5–100% (NH_4_)_2_SO_4_. After dialysis, the retentate saturated with 75% (NH_4_)_2_SO_4_ (1.412 U/mg protein) was loaded onto a DEAE-Cellulose ionic exchange column to seize five fractions ThLacc-Fis, ThLacc-S, ThLacc-T, ThLacc-Fo, and ThLacc-Fif; the second fraction, ThLacc-S, eluted with 0.3 M NaCl had the highest specific activity of 2.864 U/mg protein, with a 2.69-fold increase in purity and 59.48% yield. ThLacc-S was therefore re-chromatographed on a Sephadex G-100 gel filtration column and this generated: a 20.76-fold increase in purity and 46.79% yield, with specific activity of 22.111 U/mg protein.

**TABLE 1:** Summary of purification steps of laccase ThLacc-S from *Trametes hirsuta*.

**Purification step**	**Total activity (U/mL)**	**Total protein (mg/mL)**	**Specific activity (U/mg)**	**Purification fold**	**Yield (%)**
Crude extract	1.276	1.198	1.065	1	100
Salt precipitation	1.072	0.759	1.412	1.33	84.01
DEAE-Cellulose ionic exchange chromatography	0.759	0.265	2.864	2.69	59.48
Sephadex G-100 gel filtration chromatography	0.597	0.027	22.111	20.76	46.79

### Uniformity and Molecular Weight of ThLacc-S

**FIGURE 1:**
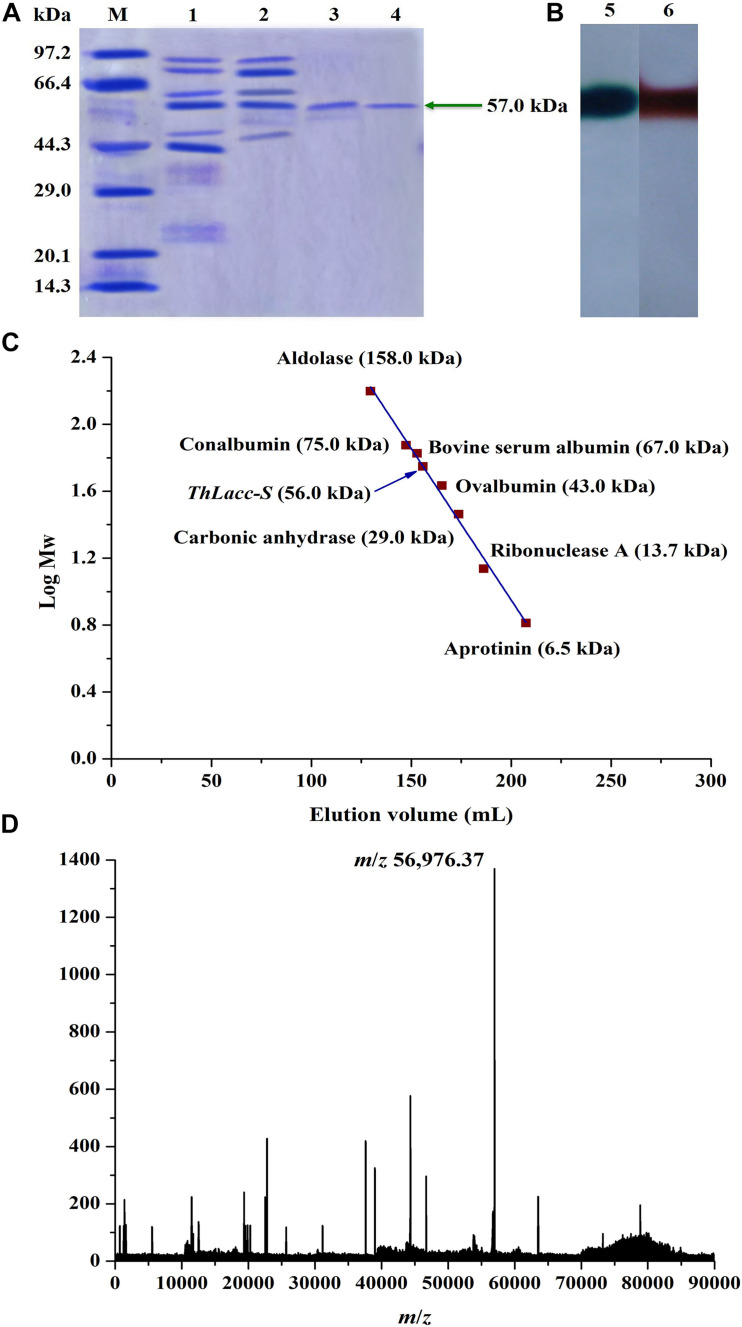
Molecular weight authentication of laccase ThLacc-S from *Trametes hirsuta*. **(A)** SDS-PAGE consisting of a 12% (w/V) separating gel (pH 8.8) and a 5% (w/V) stacking gel (pH 6.8) visualized with Coomassie Brilliant Blue R250 staining. Lane M, protein marker; lane 1, crude extract ThLacc; lane 2, laccase ThLacc purified by salt precipitation; lane 3, laccase ThLacc purified by DEAE-Cellulose ionic exchange chromatography; lane 4, laccase ThLacc purified by Sephadex G-100 gel filtration chromatography. **(B)** Native PAGE. Lane 5, ABTS staining; lane 6, guaiacol staining. **(C)** Gel filtration chromatography on a Sephadex G-100 column. **(D)** Mass spectrum acquired by MALDI-TOF MS.

Uniformity and subunit Mw of ThLacc-S were estimated by gel electrophoresis under denaturing and non-denaturing conditions. ThLacc-S emerged as a single band corresponding to an Mw of 57.0 kDa on the SDS-PAGE gel (Figure 1A). In the native PAGE spectrum stained with ABTS or guaiacol, a clear band that was responsible for laccase activity and migrated at the same Mw as in the SDS-PAGE was visualized (Figure 1B). This authenticated the monomeric nature of this enzyme and constituted a subunit Mw. Gel filtration chromatography on a Sephadex G-100 column with reference proteins supported that the native Mw of ThLacc-S was 56.0 kDa (Figure 1C). Furthermore, mass spectra from MALDI-TOF MS confirmed that ThLacc-S possessed an apparent Mw of 56976.37 Da (Figure 1D). Laccases originating from fungi exhibit single-subunit type protein structures, termed monomeric proteins, in SDS-PAGE and native PAGE gels and abundantly variable Mws that might possibly be ascribed to genetic discrepancies among different species (Claus, 2004; Baldrian, 2006; Rivera-Hoyos et al., 2013; Si et al., 2013; Mate and Alcalde, 2015; Zheng et al., 2017; Sadeghian-Abadi et al., 2019). These observations implied that ThLacc-S was a monomeric protein with an Mw of 57.0 kDa.

### Identification of ThLacc-S

**FIGURE 2:**
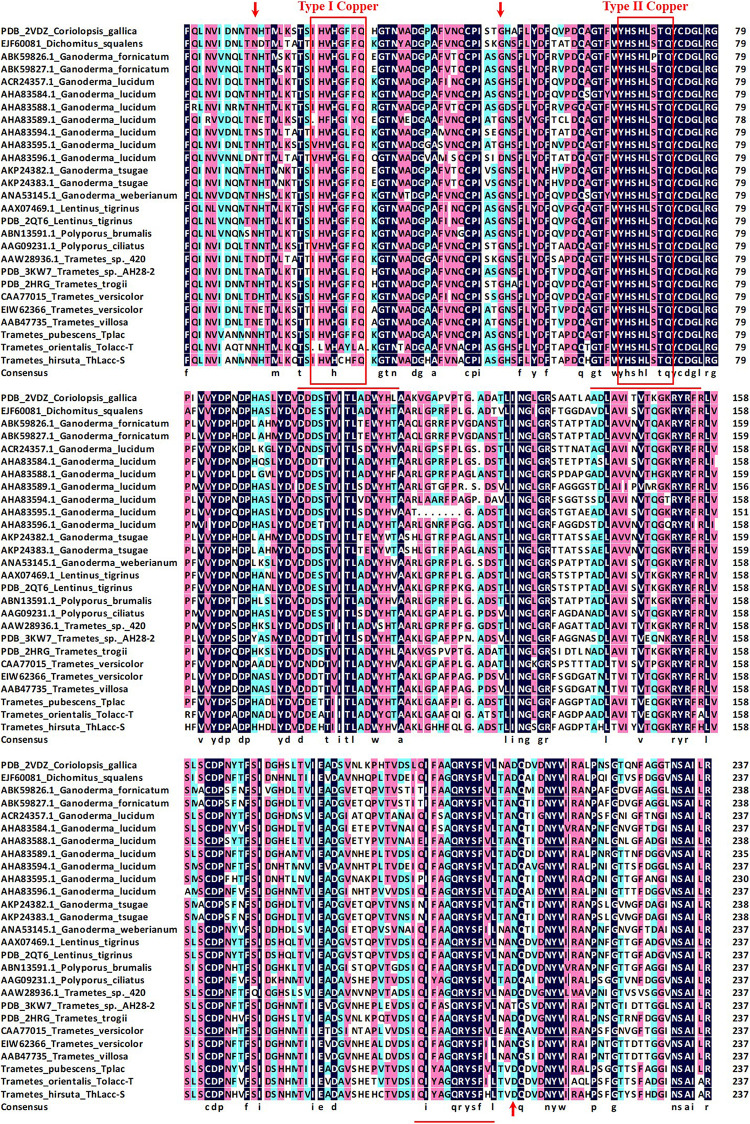
Multiple alignment of the amino acid sequence of laccase ThLacc-S from *Trametes hirsuta* with those of other fungal laccases including *Coriolopsis gallica* (PDB: 2VDZ), *Dichomitus squalens* (EJF60081), *Ganoderma fornicatum* (ABK59826.1 and ABK59827.1), *G. lucidum* (ACR24357.1, AHA83584.1, AHA83588.1, AHA83589.1, AHA83594.1, AHA83595.1, and AHA83596.1), *G. tsugae* (AKP24382.1 and AKP24383.1), *G. weberianum* (ANA53145.1), *Lentinus tigrinus* (AAX07469.1 and PDB: 2QT6), *Polyporus brumalis* (ABN13591.1), *P. ciliatus* (AAG09231.1), *Trametes* sp. 420 (AAW28936.1), *Trametes* sp. AH28-2 (PDB: 3KW7), *T. trogii* (PDB: 2HRG), *T. versicolor* (CAA77015 and EIW62366), *T. villosa* (AAB47735), *T. pubescens* Tplac, and *T. orientalis* Tolacc-T. Numbers on the right are the positions of the final amino acids in each line. Residues assumed to be involved in binding to copper are boxed in red and residues identical in all 27 sequences are highlighted with a black background. Potential glycosylation sites are indicated with red arrows. Underlined residues indicate the sequences generated through MALDI-TOF MS.

The *N*-terminal amino acid sequence of ThLacc-S (UniProt Knowledgebase accession number: C0HLV6) was acquired through trypsin digestion, ABTS staining, sequencing, and MALDI-TOF MS. Multiple alignment of the amino acid sequence of ThLacc-S with other fungal laccases (Figure 2) indicated that ThLacc-S contained representative conserved I and II copper-binding domains and shared three potential glycosylation sites (Thurston, 1994; Claus, 2004). The alignment also revealed that the *N*-terminal amino acid sequence of ThLacc-S shared sequence identity with laccases from other species of the genus *Trametes*, attaining 83.61 and 82.85% sequence similarity with Tplac from *Trametes pubescens* and Tolacc-T from *Trametes orientalis*, respectively. Moreover, the sequence of ThLacc-S harbored similarities with other laccases, including 75.63% sequence similarity with *Lentinus tigrinus* AAX07469.1 and *Polyporus ciliatus* AAG09231.1, 75.21% with *L. tigrinus* PDB: 2QT6, 74.79% with *Polyporus brumalis* ABN13591.1, 73.11% with *Trametes versicolor* CAA77015, 72.69% with *Ganoderma lucidum* AHA83584.1, 71.85% with *Trametes trogii* PDB: 2HRG and *Dichomitus squalens* EJF60081, 71.43% with *Coriolopsis gallica* PDB: 2VDZ, 70.59% with *T. versicolor* EIW62366, *Ganoderma weberianum* ANA53145.1, *G. lucidum* AHA83595.1 and ACR24357.1, and *Trametes villosa* AAB47735, 70.17% with *Trametes* sp. 420 AAW28936.1, 69.33% with *Trametes* sp. AH28-2 PDB: 3KW7, 68.49% with *G. lucidum* AHA83594.1, 68.20% with *G. lucidum* AHA83588.1, 68.07% with *G. lucidum* AHA83589.1, 67.65% with *G. lucidum* AHA83596.1, 65.69% with *Ganoderma fornicatum* ABK59827.1, 64.85% with *Ganoderma tsugae* AKP24383.1 and AKP24382.1, and 64.44% with *G. fornicatum* ABK59826.1, respectively. This indicated that ThLacc-S from *T. hirsuta* was a novel member of the laccase family.

### Effect of pH and Temperature on ThLacc-S Activity and Stability

**FIGURE 3:**
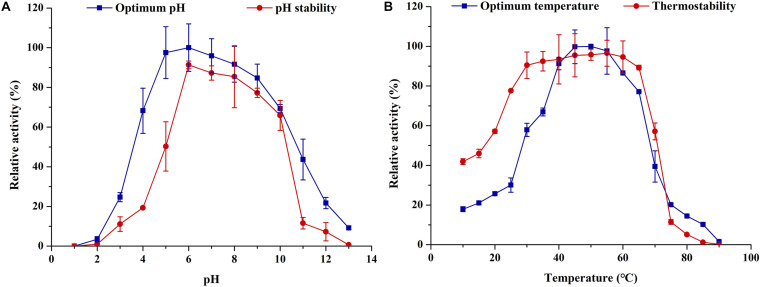
**(A)** Optimum pH and **(B)** thermostability of laccase ThLacc-S from *Trametes hirsuta*.

ThLacc-S was ≥ 60% active over a wide pH range from 4.0 to 10.0, with maximum activity (0.612 U/mL) at pH 6.0 (Figure 3A); the enzyme was more active in neutral and alkaline pH. There was a sharp decline in laccase activity at pH < 4.0 or > 10.0, presumably attributed to enzyme denaturation or inactivation. Congruent with the activity data, ThLacc-S stability was higher within the pH range of 5.0–10.0 compared with pH outside this range, as evidenced by preservation of enzyme activity above 50% after incubation at pH 5.0–10.0 for 72 h. The wide pH tolerance of this enzyme could be explained by increases in charges of amino acid residues within the active site (Claus, 2004; Pezzella et al., 2015).

Of the tested temperatures (10–90°C) depicted in Figure 3B, the range 30–65°C was beneficial for ThLacc-S as the enzyme maintained more than 57% of its activity at these temperatures. The optimum temperature for ThLacc-S activity was 50°C, equivalent to specific activity of 2.037 U/mL. Incubation at < 25°C or > 70°C dramatically reduced activity and stability of ThLacc-S. However, comparable marked improvements in ThLacc-S activity were observed after incubation for 2 h at 10–40 and 60–70°C. The activity and stability of this enzyme, as well as the reduced sensitivity to extreme temperatures, are superior to those of other previously described laccases (Rivera-Hoyos et al., 2013; Sadeghian-Abadi et al., 2019).

Hence, one important feature of ThLacc-S that is applicable to numerous industrial and biotechnological areas operating under severe conditions, is the thermo-alkali stability across considerably abundant pH and temperature ranges for a prolonged duration.

### Effect of Metallic Ions, Specific Inhibitors, Surfactants, and Organic Solvents on ThLacc-S Activity

The impact of metallic ions, specific inhibitors, surfactants, and organic solvents on ThLacc-S activity are outlined in Table 2.

**TABLE 2:** Effect of metallic ions, specific inhibitors, surfactants, and organic solvents on activity of laccase ThLacc-S from *Trametes hirsuta*.

**Additive**		**Concentration**	**Relative activity (%)**
Control	—	—	100 ± 0.30
Metallic ion	Li^+^	25.0 mM	89.95 ± 4.08 jklm
	Na^+^	25.0 mM	173.39 ± 4.24 de
	Mg^2+^	25.0 mM	252.56 ± 5.74 ab
	Al^3+^	25.0 mM	69.63 ± 4.60 op
	K^+^	25.0 mM	244.13 ± 12.60 b
	Ca^2+^	25.0 mM	51.06 ± 1.30 qr
	Mn^2+^	25.0 mM	126.98 ± 12.22 gh
	Fe^2+^	25.0 mM	159.66 ± 8.12 ef
	Fe^3+^	25.0 mM	11.96 ± 1.53 tuv
	Ni^2+^	25.0 mM	177.96 ± 9.86 d
	Cu^2+^	25.0 mM	261.97 ± 11.43 a
	Zn^2+^	25.0 mM	217.71 ± 5.89 c
	Pb^2+^	25.0 mM	144.02 ± 10.07 fg
	Ag^+^	25.0 mM	101.49 ± 7.60 ijk
	Cd^2+^	25.0 mM	220.42 ± 7.64 c
	Ba^2+^	25.0 mM	24.68 ± 1.77 stu
	Hg^2+^	25.0 mM	2.83 ± 1.61 v
Specific inhibitor	IAA (cysteine protease inhibitor)	2.0 mM	Not detected
		5.0 mM	Not detected
		10.0 mM	Not detected
	TLCK (lysine protease inhibitor)	2.0 mM	100 ± 2.94 jk
		5.0 mM	100 ± 3.01 jk
		10.0 mM	99.17 ± 8.55 jk
	PMSF (serine protease inhibitor)	2.0 mM	Not detected
		5.0 mM	Not detected
		10.0 mM	Not detected
	DEP (histidine protease inhibitor)	2.0 mM	Not detected
		5.0 mM	Not detected
		10.0 mM	Not detected
	NAI (tyrosine protease inhibitor)	2.0 mM	Not detected
		5.0 mM	Not detected
		10.0 mM	Not detected
	Pepstatin A (aspartate protease inhibitor)	2.0 mM	100 ± 5.83 jk
		5.0 mM	100 ± 10.24 jk
		10.0 mM	99.84 ± 8.64 jk
	EDTA (cheating agent)	2.0 mM	45.67 ± 2.05 qr
		5.0 mM	26.54 ± 2.49 st
		10.0 mM	7.76 ± 0.31 uv
Surfactant	SDS (ionic surfactant)	2.0 mM	75.50 ± 7.62 mnop
		5.0 mM	62.46 ± 4.99 pq
		10.0 mM	36.21 ± 3.10 rs
	Triton X-100 (non-ionic surfactant)	10% (V/V)	103.42 ± 8.58 ij
		30% (V/V)	117.79 ± 3.41 hi
Organic solvent	Methanol	10% (V/V)	95.32 ± 7.12 jkl
		30% (V/V)	81.43 ± 7.95 lmno
	Ethanol	10% (V/V)	90.54 ± 6.67 jklm
		30% (V/V)	84.32 ± 0.27 klmno
	Propanol	10% (V/V)	87.47 ± 2.45 jklmn
		30% (V/V)	79.56 ± 8.17 lmnop
	Hexane	10% (V/V)	92.78 ± 4.99 jklm
		30% (V/V)	76.26 ± 5.01 mnop
	Acetone	10% (V/V)	62.18 ± 3.24 pq
		30% (V/V)	50.06 ± 4.69 qr
	Toluene	10% (V/V)	135.05 ± 8.68 gh
		30% (V/V)	167.98 ± 12.67 de
	Chloroform	10% (V/V)	85.37 ± 5.69 klmno
		30% (V/V)	70.51 ± 7.68 nop

*Data are mean ± standard deviation. Different lowercase letters represent significant differences at P < 0.05 level by Waller-Duncan test.*

Laccase activity relative to the control was 261.97, 252.56, 244.13, 220.42, 217.71, 177.96, 173.39, 159.66, 144.02, or 126.98%, following separate addition of 25.0 mM Cu^2+^, Mg^2+^, K^+^, Cd^2+^, Zn^2+^, Ni^2+^, Na^+^, Fe^2+^, Pb^2+^, or Mn^2+^, respectively. This indicated that ThLacc-S requires these metallic ions to retain the conformation of its active site. The greatest effect of Cu^2+^ on laccase activity might be owing to this ion being involved in the catalytic process, since typical laccases are known to contain three types of copper sites and a core center with a cluster of four copper atoms (Thurston, 1994; Claus, 2004). Other metallic ions like Li^+^, Al^3+^, Ca^2+^, Ba^2+^, or Fe^3+^ inhibited ThLacc-S activity possibly due to binding near the T1 site, which blocks access of substrates to the site and thus the ions act as competitive inhibitors for electron donors (Thurston, 1994; Si et al., 2013). Obviously, Hg^2+^ strongly inhibited ThLacc-S activity and is presumed to be a key inactivator of ThLacc-S as this metallic ion can react with sulfhydryl groups present on histidine residues of the catalytic site and displace other active metallic ions from their binding positions; such competitive binding results in enzymatic deactivation (Claus, 2004; Yahiaoui et al., 2019).

Protease inhibitors are efficient tools for delimiting categories of enzymes (Powers et al., 2002). IAA (cysteine protease inhibitor), PMSF (serine protease inhibitor), DEP (histidine protease inhibitor), and NAI (tyrosine protease inhibitor) abolished ThLacc-S activity at 2.0, 5.0, and 10.0 mM, respectively. This indicated that cysteine, serine, histidine, and tyrosine residues were present in the functional site of this enzyme. Complete inhibition by IAA also supported that sulfhydryl groups are required to retain the structural conformation of ThLacc-S, as evidenced by Hg^2+^ being a key inactivator of this enzyme. There were negligible changes in ThLacc-S activity after incubation with TLCK (lysine protease inhibitor) or pepstatin A (aspartate protease inhibitor), indicating that ThLacc-S was not a lysine or aspartate protease. The chelator EDTA at 2.0, 5.0, and 10.0 mM resulted in gradual decreases in ThLacc-S activity to 45.67, 26.54, and 7.76%, respectively, expounding that metallic ions are involved in enzymatic catalysis by ThLacc-S, and therefore ThLacc-S is a rare metalloenzyme.

Surfactants, including ionic and non-ionic types, are reported to influence enzymatic conformation by interacting with charges on the surface of the enzyme (Shao et al., 1993). SDS, a recognized ionic surfactant, interfered with ThLacc-S activity at all assayed concentrations (2.0, 5.0, and 10.0 mM), with diminished activities of 75.50, 62.46, and 36.21%, respectively. A slight promotion of ThLacc-S activity was observed in the presence of 10 or 30% (V/V) non-ionic surfactant Triton X-100. This is attributed to Triton X-100 preventing formation of self-aggregates of ThLacc-S and stabilizing folding of the enzyme.

In the presence of organic solvents, ThLacc-S was strongly activated by toluene, attaining approximately 1.68-fold laccase activity compared with the control. Over 70% of ThLacc-S activity was preserved by separate supplements of ethanol, methanol, propanol, hexane, and chloroform, respectively, whereas acetone reduced laccase activity by almost 50% compared with the control. These findings endorsed that when encountering organic solvents, enzymatic activity is affected by the distribution of water molecules and characteristics of organic solvents like hydrophobicity and polarity, in addition to the conformation of the enzyme itself (Wan et al., 2010).

The observed enhancement and maintenance of ThLacc-S activity demonstrate the extraordinary tolerance of this novel enzyme toward metallic ions, surfactants, and organic solvents, and render it a promising catalyst to suffice industrial demands.

### Kinetic Parameters of ThLacc-S

A Lineweaver-Burk plot relating reaction velocity of ThLacc-S to ABTS concentrations can be seen in Supplementary Figure 1. Kinetic parameters *K*_*m*_ and *k*_*cat*_/*K*_*m*_ of ThLacc-S against the substrate ABTS were calculated to be 87.466 μM and 1.479 s^–1^μM^–1^, respectively. Comparisons of the kinetic parameters of ThLacc-S with those of other reported laccases are shown in Table 3. The lower *K*_*m*_ and higher *k*_*cat*_/*K*_*m*_ values compared with the values for the other laccases affirmed the strong affinity and reaction velocity of this enzyme toward the substrate (Rivera-Hoyos et al., 2013; Yang et al., 2020). This kinetic behavior may be dependent on genetic diversity of the different laccase producing sources as well as the nature and structure of the enzyme (Rivera-Hoyos et al., 2013; Kelbert et al., 2021).

**TABLE 3:** Comparisons of the kinetic parameters of laccase ThLacc-S from *Trametes hirsuta* with those of other reported laccases.

**Laccase producing source**	**Name**	***K*_*m*_ (μM)**	***k*_*cat*_ (s^–1^)**	***k*_*cat*_/*K*_*m*_ (s^–1^μM^–1^)**	**References**
*Trametes hirsuta*	ThLacc-S	87.466	129.367	1.479	This study
*Agaricus bisporus* CU13	Lacc1	0.394	-	-	Othman et al., 2018
	Lacc2	0.158	-	-	
*Cerrena unicolor* GSM-01	CUL	302.7	286.5	0.946	Wang et al., 2017
*Ganoderma lucidum*	-	47	54	1.149	Manavalan et al., 2013
*Lentinus strigosus* 1566	Laccase I	11.0	460.97	41.906	Kolomytseva et al., 2019
	Laccase II	17.0	141.02	8.295	
	Laccase III	263.2	522.38	1.985	
*Marasmius* sp.	Laccase-related enzyme I	3.9	120	30.769	Schückel et al., 2011
*Oudemansiella canarii* EF72	-	46.18	-	-	Iark et al., 2019
*Pleurotus ostreatus* HAUCC 162	rLACC6	459	81.35	0.177	Zhuo et al., 2018
	rLACC9	413	20.10	0.049	
	rLACC10	43	15.50	0.360	
*Pycnoporus coccineus* BRFM 938	BRFM 938 laccase	26	218.1	8.388	Uzan et al., 2010
*Pycnoporus sanguineus* BRFM 902	BRFM 902 laccase	32	236.9	7.403	
*Py. sanguineus* BRFM 66	BRFM 66 laccase	33	214.3	6.494	
*Thielavia* sp.	TaLac1	23.70	4.14	0.175	Mtibaà et al., 2018
*Trametes orientalis*	Tolacc-T	333.3	21.81	0.065	Zheng et al., 2017
*Trametes pubescens* Cui 7571	Tplac	105.0	876	8.343	Si et al., 2013
*Trametes trogii* BAFC 463	LCC3	250	399	1.596	Campos et al., 2016
*Tr. trogii* S0301	Lac 37 II	16.1	2977	184.907	Yang et al., 2020
*Trametes versicolor*	LC	4.05	-	-	Kelbert et al., 2021
*Trametes* sp. LAC-01	LAC-01	30.28	-	-	Ling et al., 2015
*Trametes* sp. F1635	TsL	18.58	-	-	Wang et al., 2018

*All data were obtained by using ABTS as substrates.*

### EDC-Bioremediating Capacity of ThLacc-S

Partial metabolic by-products of E2 in the ThLacc-S-mediated reaction process were identified through GC-MS analysis and comprised estrone (E1), 2-OH-E2, 4-OH-E1, 2-OH-E1, 2-OH-E2-OCH_3_, E2-BP1, E2-BP2, E2-BP3, and E2-BP4 (Table 4 and Supplementary Table 1). This finding was in accordance with a previous study reporting similar E2 metabolites mediated by the *T. versicolor* laccase (Liu et al., 2021). These data substantiated that ThLacc-S could efficiently catalyze the transformation of dominant, highly toxic, natural estrogen such as E2 in the absence of redox mediators, with formation of corresponding phenoxy radical intermediates, accompanied by four-electron reduction of molecular oxygen to water. In view of the detected by-products and pertinent literatures (Ye et al., 2017; Li et al., 2020; Wang et al., 2020), different metabolic pattern of E2 form products with additional hydroxyl and methoxyl groups to E2 and E1, and the degradation routes by ThLacc-S were deduced to be accomplished by oxidation, hydroxylation, carboxylation, dehydrogenation, dehydroxylation, demethylation, and methoxylation. Particularly noticeable are studies showing that E2 metabolized by methoxylation can hinder formation of its quinone form that are major carcinogenic metabolites (Cavalieri and Rogan, 2011). These results marked that this type of metabolism was also admitted to be a detoxification behavior and suggested that ThLacc-S would be an effective, safe, and green catalyst for various industrial applications, especially those involving bioremediation and biodegradation. Further investigations are necessary to elucidate the detailed mechanism of E2 metabolism by laccase, to explore estrogenic activities of the intermediates, and to determine the laccase functionality at more environmentally relevant concentrations of EDCs.

**TABLE 4:** Partial metabolic by-products of 17β-estradiol (E2) identified through GC-MS in the reaction process mediated by laccase ThLacc-S from *Trametes hirsuta*.

**Compound**	***m*/*z***	**Chemical formula**	**Deduced chemical structure**
E2	272.97	C_1__8_H_2__4_O_2_	
			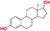
Estrone (E1)	271.06	C_1__8_H_2__2_O_2_	
			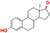
2-OH-E2	288.31	C_1__8_H_2__4_O_3_	
			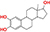
4-OH-E1	286.36	C_1__8_H_2__2_O_3_	
			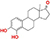
2-OH-E1	286.74	C_1__8_H_2__2_O_3_	
			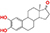
2-OH-E2-OCH_3_	317.95	C_1__9_H_2__6_O_4_	
			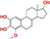
E2-BP1	318.31	C_1__8_H_2__2_O_5_	
			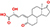
E2-BP2	335.12	C_1__8_H_2__2_O_6_	
			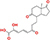
E2-BP3	351.05	C_1__8_H_2__2_O_7_	
			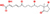
E2-BP4	369.94	C_1__7_H_2__2_O_9_	
			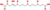

## Conclusion

This study identified an efficient laccase, ThLacc-S, from white rot fungus *T. hirsuta* that could be competent with multifarious hardness because it can tolerate wide ranges of thermo-alkali conditions and is active in the presence of diverse metallic ions, surfactants, and organic solvents. Furthermore, this enzyme proved to be a robust, eco-friendly, bioremediating agent for E2 removal. The findings of this study not only enrich those of existing laccases as emerging, environmentally safe candidates for industrial applications such as bioremediation, but also provide new insights into the functional involvement of laccases in the metabolism of EDCs.

## Data Availability Statement

The datasets presented in this study can be found in online repositories. The names of the repository/repositories and accession number(s) can be found below: UniProt Knowledgebase, accession no: C0HLV6; NCBI, accession no: MW881532.

## Author Contributions

JS and BC conceived and designed experiments. JS, HM, and YC performed experiments. JS, BC, and YD wrote the manuscript. All authors reviewed the manuscript before submission.

## Conflict of Interest

The authors declare that the research was conducted in the absence of any commercial or financial relationships that could be construed as a potential conflict of interest.
