# Supplementary material for: Introducing a Thermo-Alkali-Stable, Metallic Ion-Tolerant Laccase Purified From White Rot Fungus Trametes hirsuta
Source: Front Microbiol. 2021 May 21;12:670163. doi: 10.3389/fmicb.2021.670163 (PMC8176223; doi:10.3389/fmicb.2021.670163)
Supplement: Supplementary file 1 [file Data_Sheet_1.doc]

**Supplementary Material**


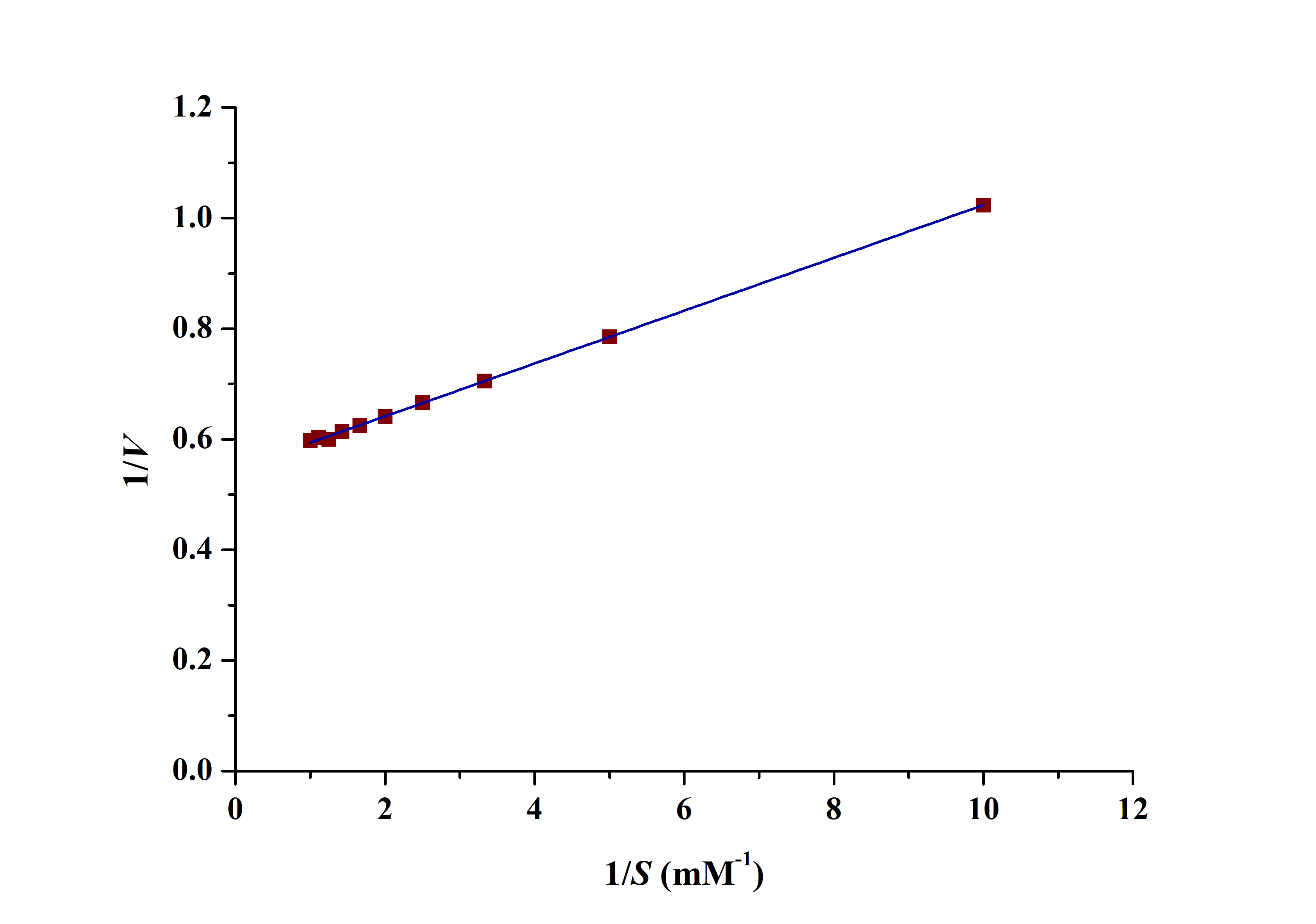


**SUPPLEMENTARY FIGURE 1.** A Lineweaver-Burk plot relating reaction velocity of laccase ThLacc-S from *Trametes hirsuta* to ABTS concentrations.

**SUPPLEMENTARY TABLE 1.** GC-MS spectral data of the partial metabolic by-products of 17*β*-estradiol (E2) in the reaction process mediated by laccase ThLacc-S from *Trametes hirsuta*.

| **Compound** | **Mass spectra** |
| --- | --- |
| E2 | 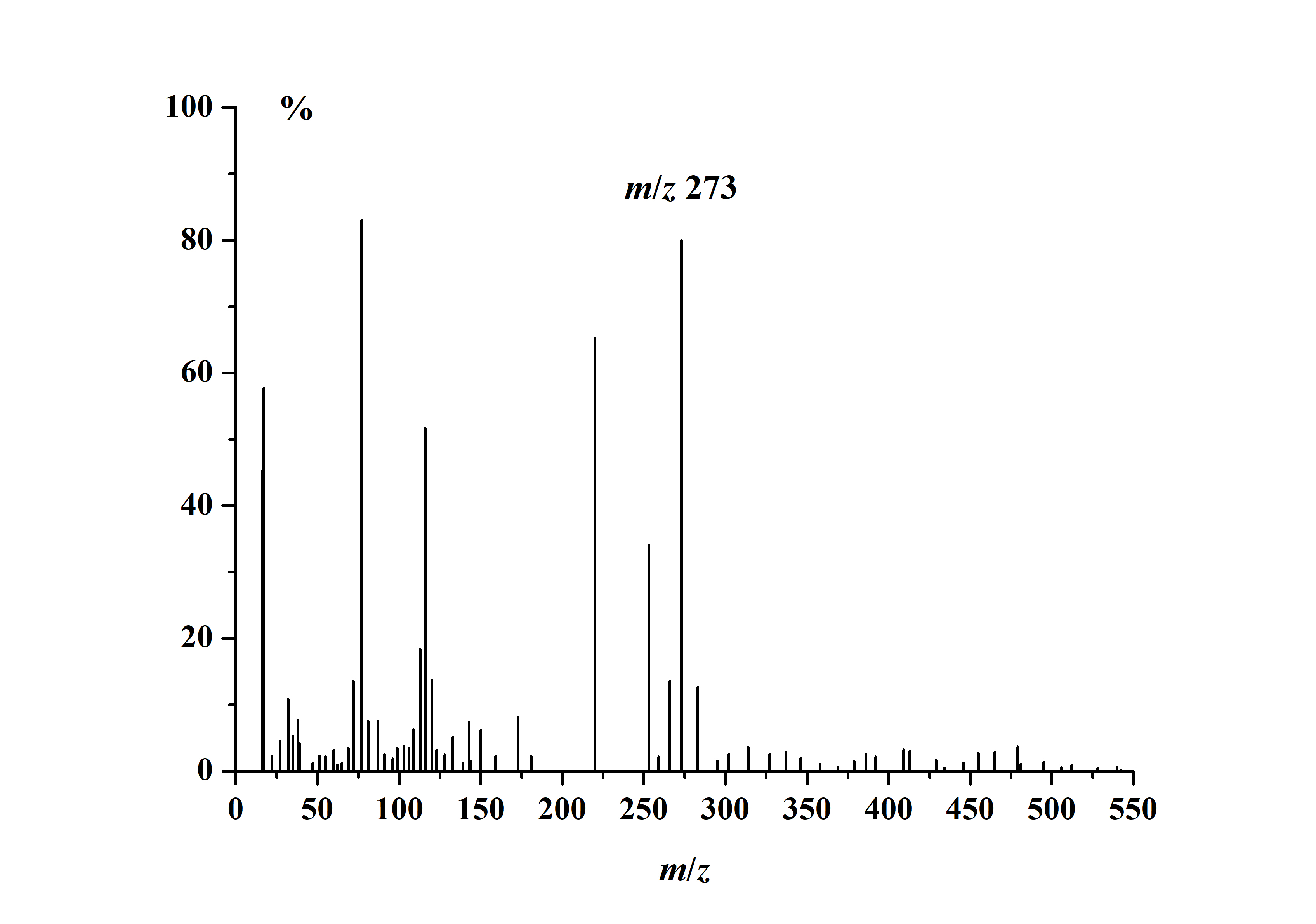 |
| Estrone (E1) | 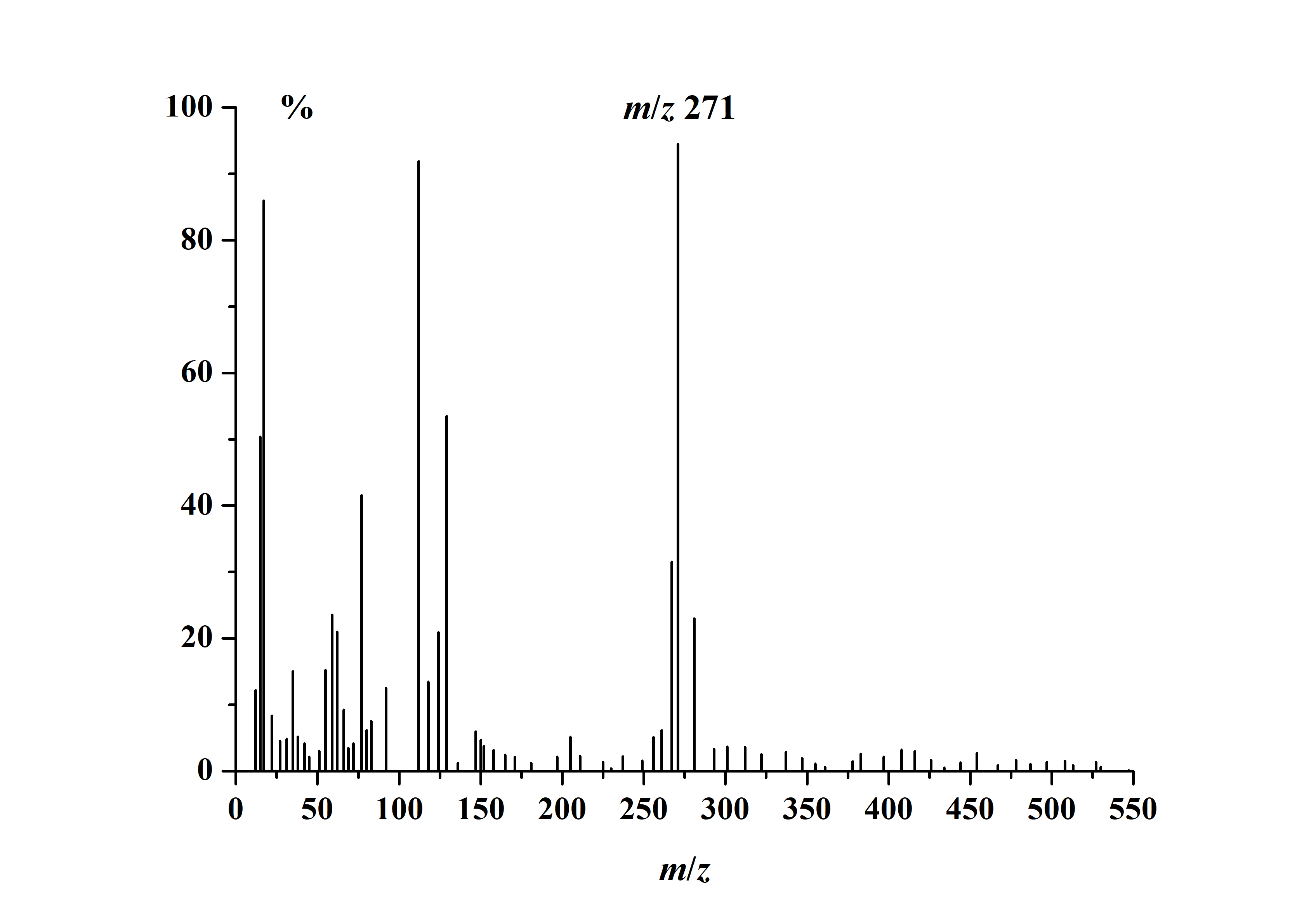 |
| 2-OH-E2 | 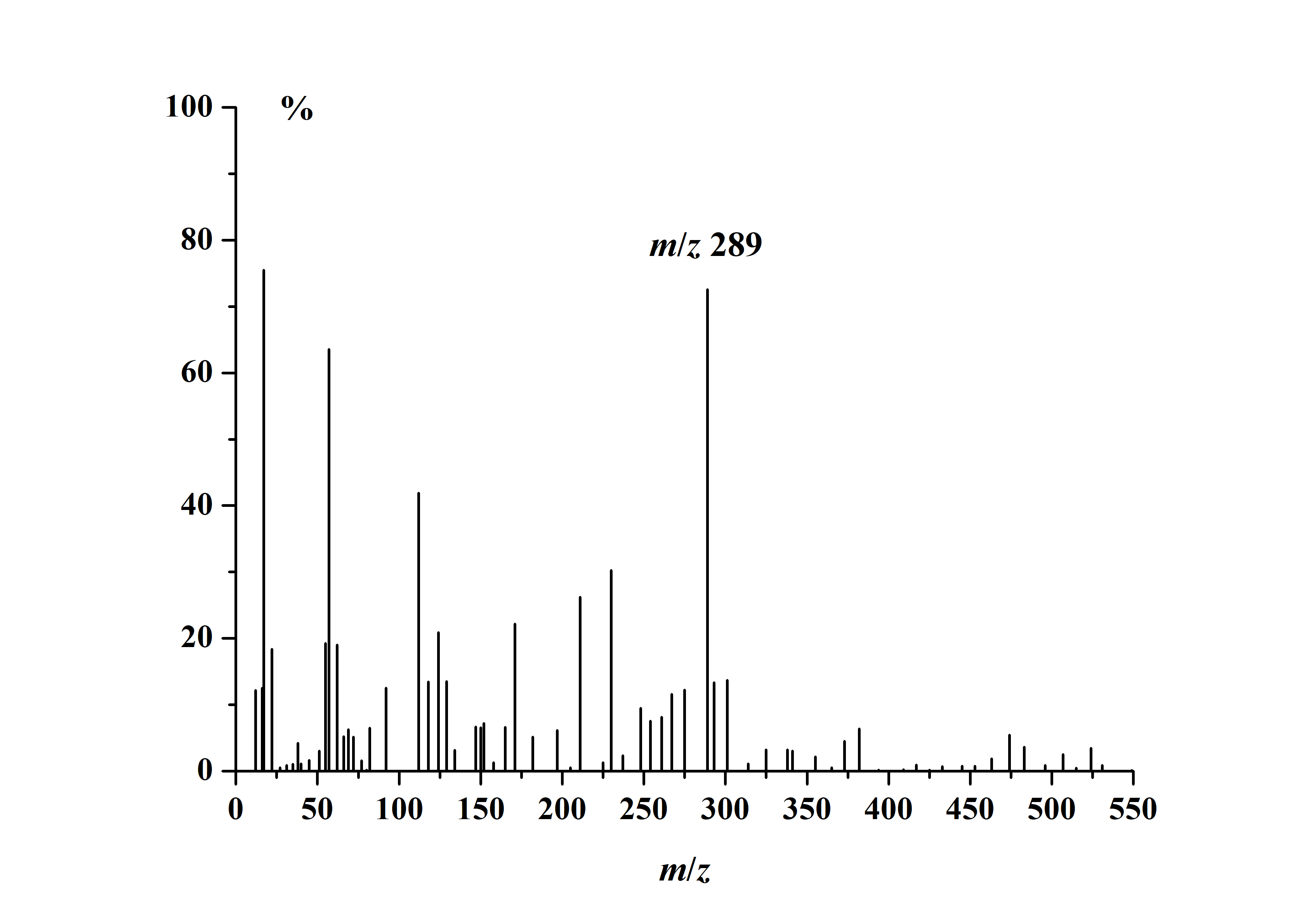 |
| 4-OH-E1 | 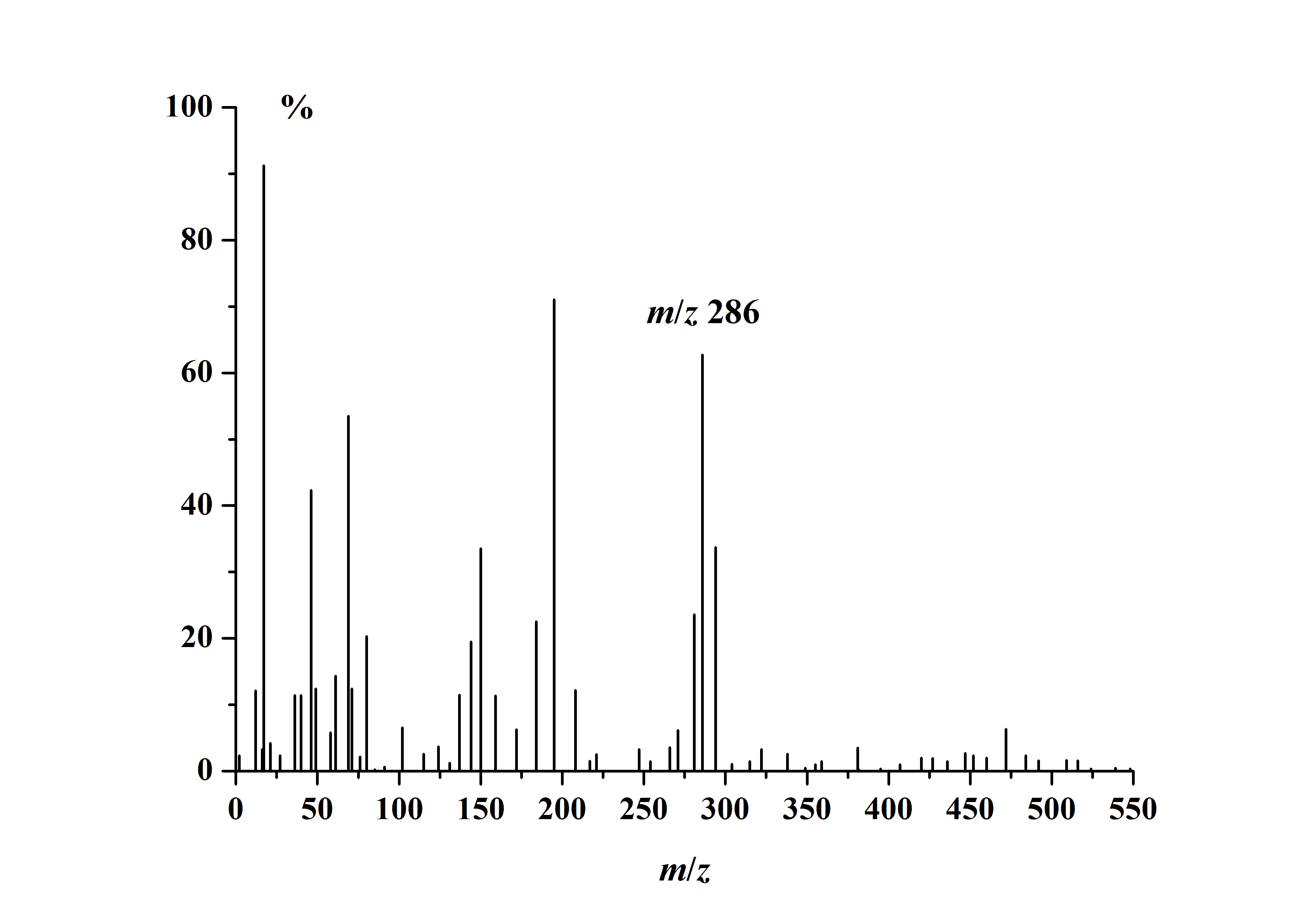 |
| 2-OH-E1 | 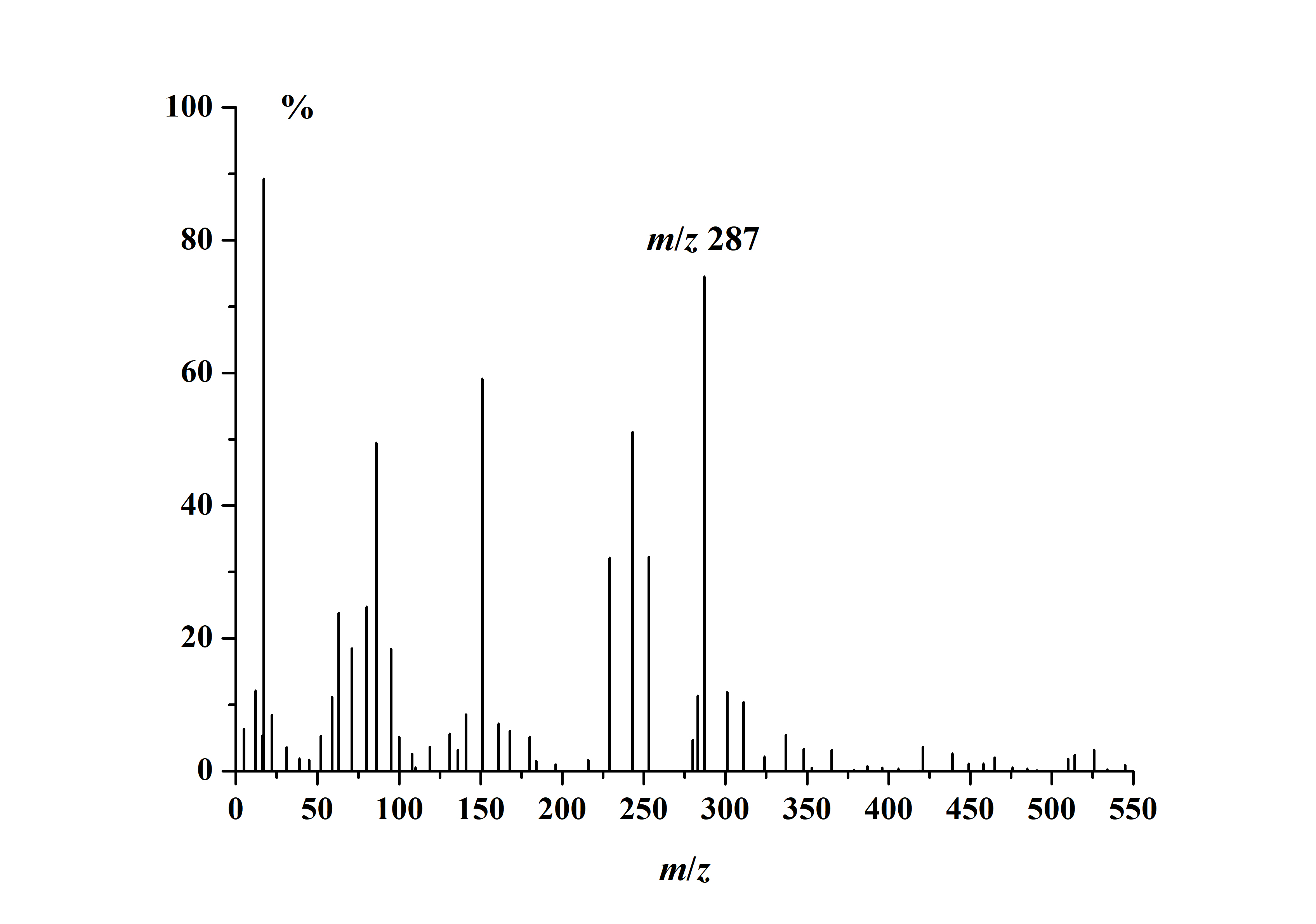 |
| 2-OH-E2-OCH_3_ | 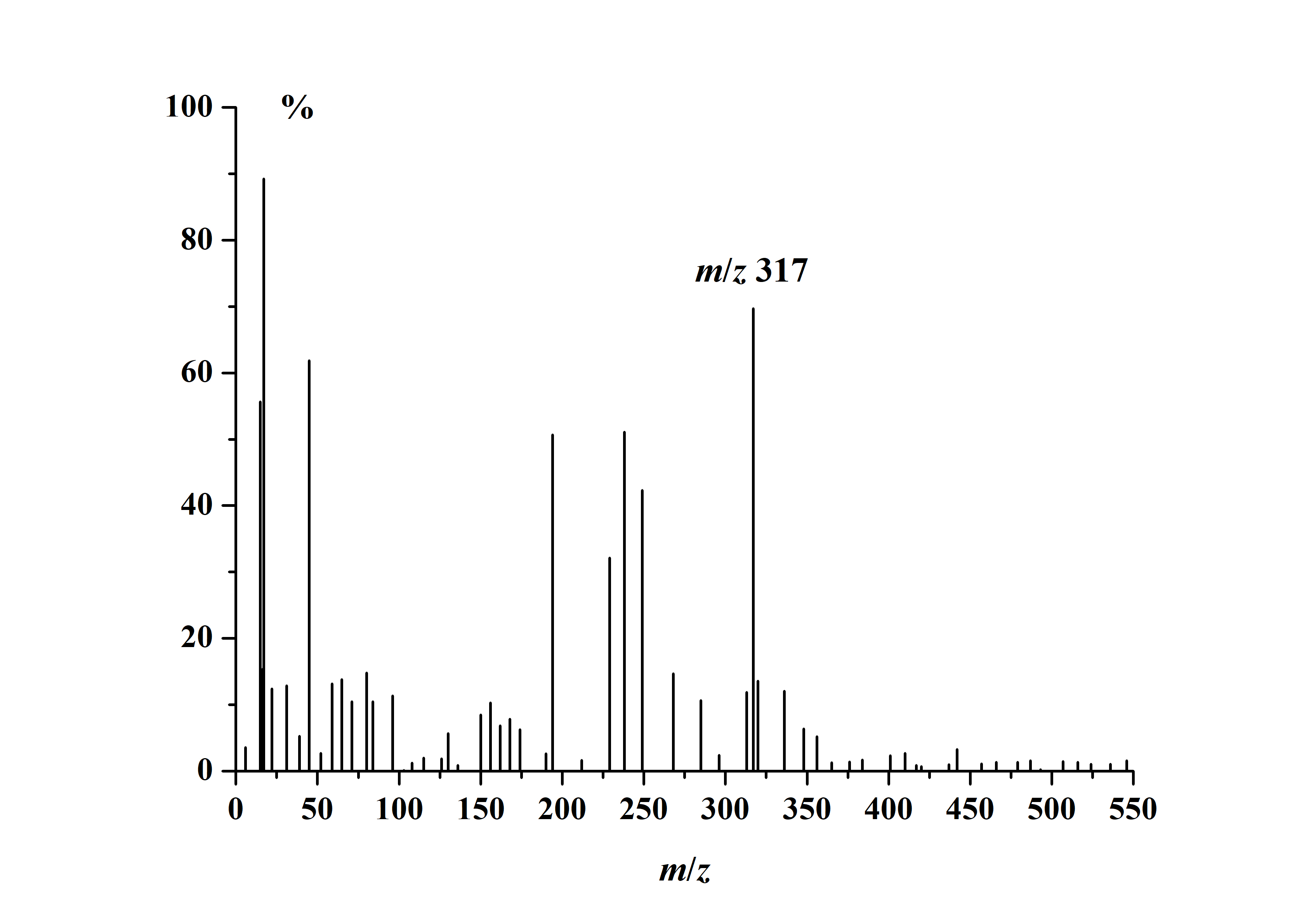 |
| E2-BP1 | 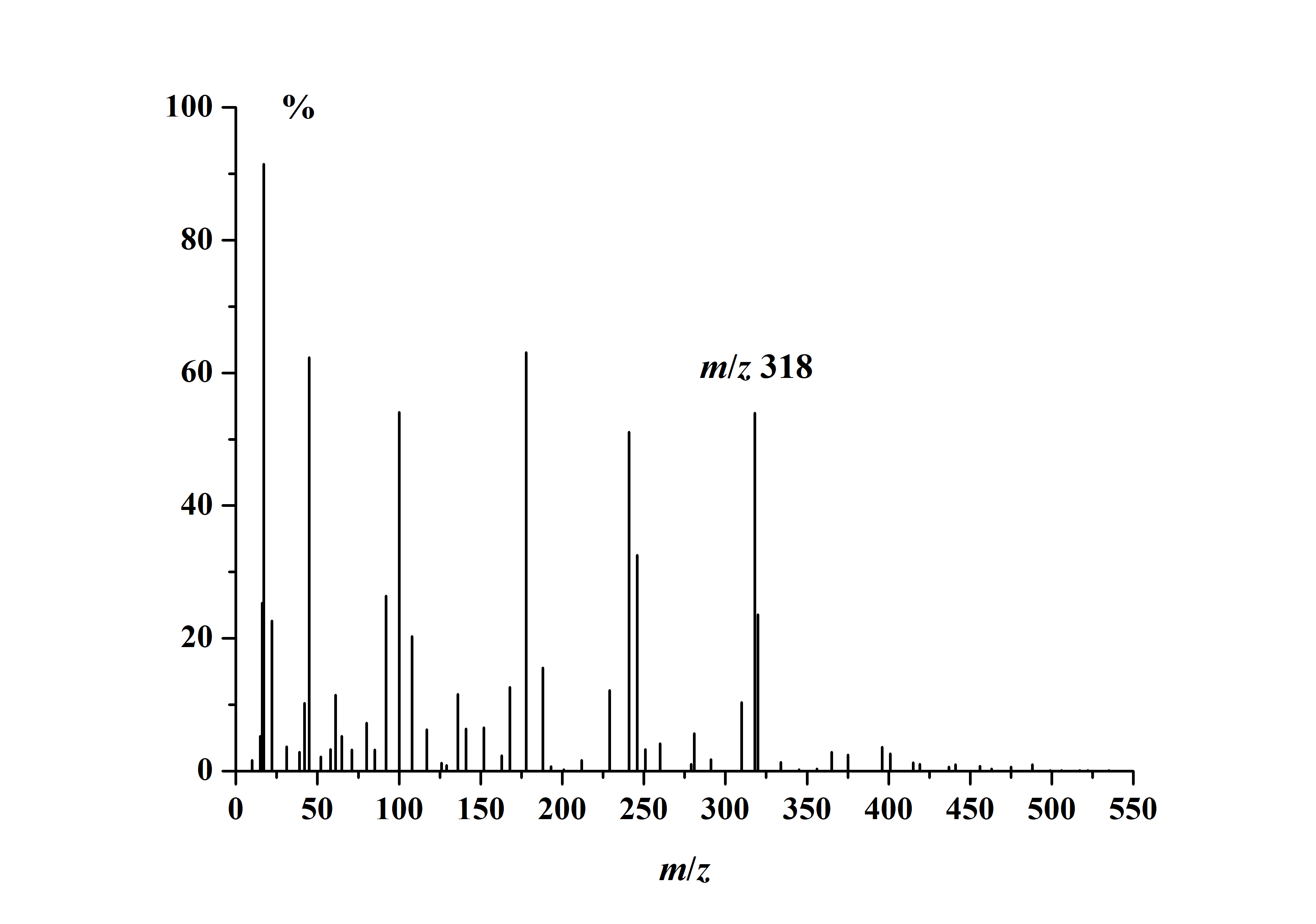 |
| E2-BP2 | 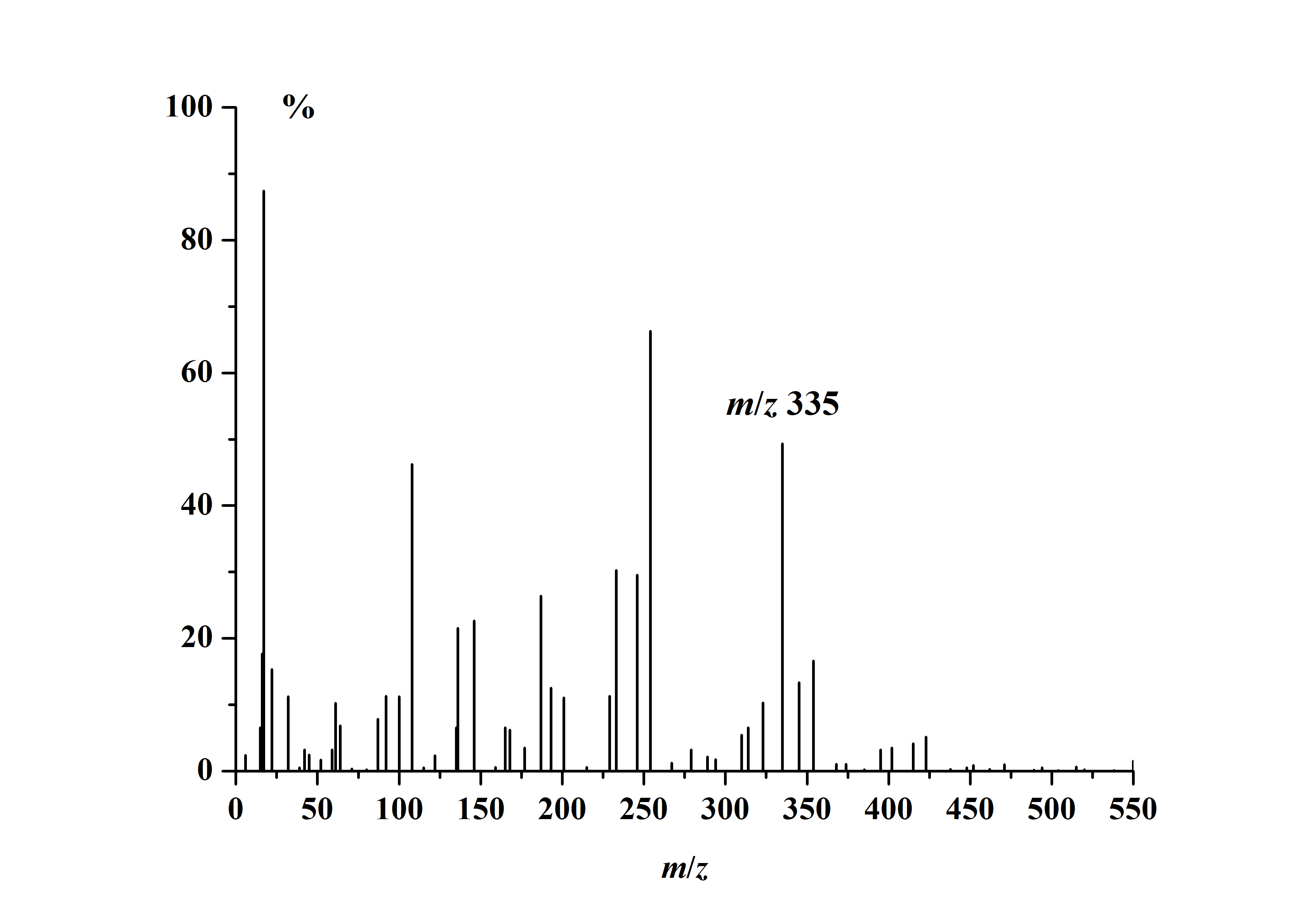 |
| E2-BP3 | 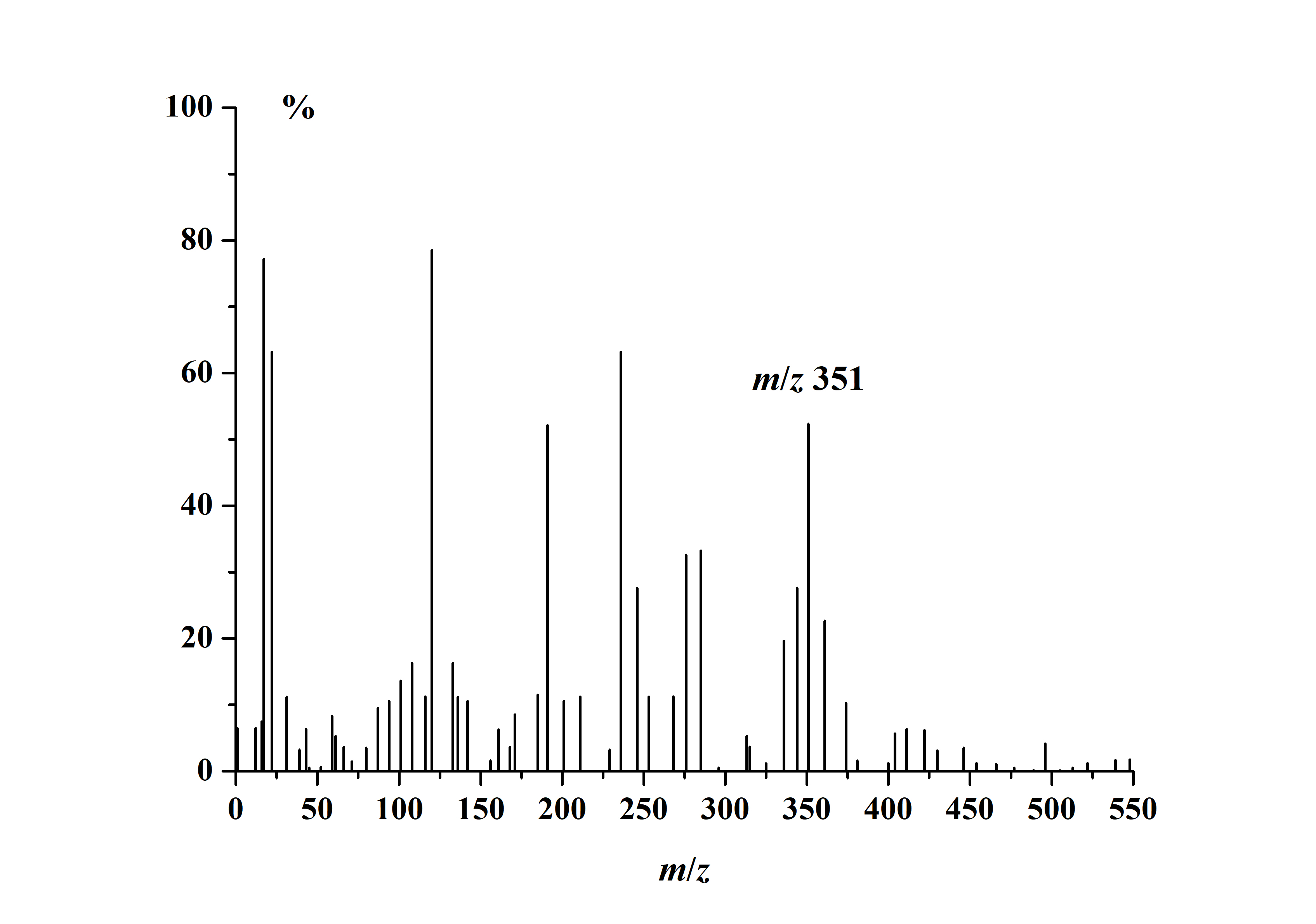 |
| E2-BP4 | 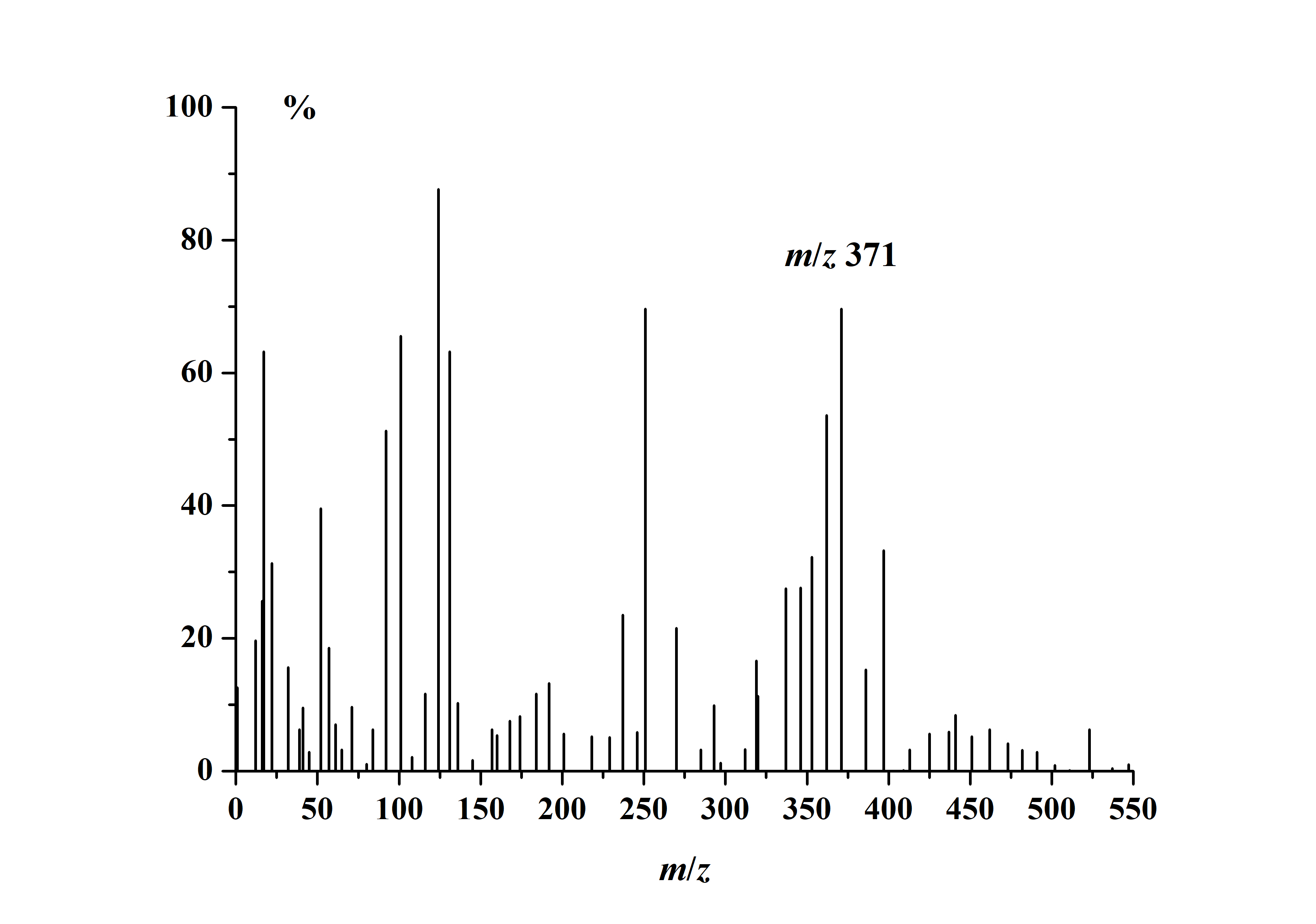 |
